# Focal central neurological disorder in patients with autoimmune thyroid disease without encephalopathy

**DOI:** 10.1002/ccr3.8519

**Published:** 2024-02-08

**Authors:** Parisa Sharifi, Sepideh Paybast, Abdorreza Naser Moghadasi

**Affiliations:** ^1^ Multiple Sclerosis Research Center, Neuroscience Institute Tehran University of Medical Sciences Tehran Iran; ^2^ Department of Neurology, Imam Hosein Educational Hospital, School of Medicine Shahid Beheshti University of Medical Sciences Tehran Iran

**Keywords:** autoimmune thyroid disease, focal central neurological disorder, Hashimoto's encephalopathy, Hashimoto's thyroiditis

## Abstract

The report underscores the necessity for a comprehensive evaluation in patients with suggestive laboratory findings or AITD history. Prompt diagnosis and appropriate management are imperative in averting long‐term complications.

## 1 INTRODUCTION

Autoimmune thyroid disease (AITD), a common autoimmune disorder primarily affecting women, is characterized by the presence of anti‐thyroid antibodies and infiltration of lymphocytes in the thyroid gland.^1^ The clinical presentation of AITD can vary depending on the stage and severity of the disease. It may include fatigue, weight gain, sensitivity to cold, dry skin, hair loss, and joint pain, with neurological symptoms occasionally manifesting, such as peripheral neuropathy, myopathy, ataxia, encephalopathy, headache, and depression.^2^ Hashimoto's encephalopathy (HE) refers to an encephalopathy with an autoimmune cause and high anti‐thyroid peroxidase antibody titers.^3^ Herein, we present two cases of young female individuals diagnosed with AITD who presented to our clinic with focal neurological disorders with no neurological symptoms of HE.

## 2 CASE PRESENTATION

### 2.1 Case 1

A 39‐year‐old female patient was admitted to our hospital complaining of headaches, pain, and paralysis in the left hemifacial region, along with diplopia. The patient had previously experienced pain and diplopia and was admitted to another healthcare facility where treatment with corticosteroids resulted in partial clinical improvement. Following discharge, the patient reported a recurrence of symptoms after tapering off the corticosteroids, albeit with a milder presentation. 3 days prior to her most recent admission, the patient's symptoms worsened, prompting her to seek medical attention at our institution. Notably, the patient denied experiencing any sensory or motor dysfunction or symptoms related to infectious diseases. Her headaches were persistent throughout the day and intensified with positional changes. On physical examination, the patient exhibited no dysarthria, and her pupils were normal in size, with a normal reaction to light. Additionally, her eye movements were regular in all directions, with no observed ptosis, lid lag, or nystagmus. She suffered from left‐sided facial plegia. No evidence of meningeal irritation was observed, and other clinical examinations were unremarkable.

**FIGURE 1:**
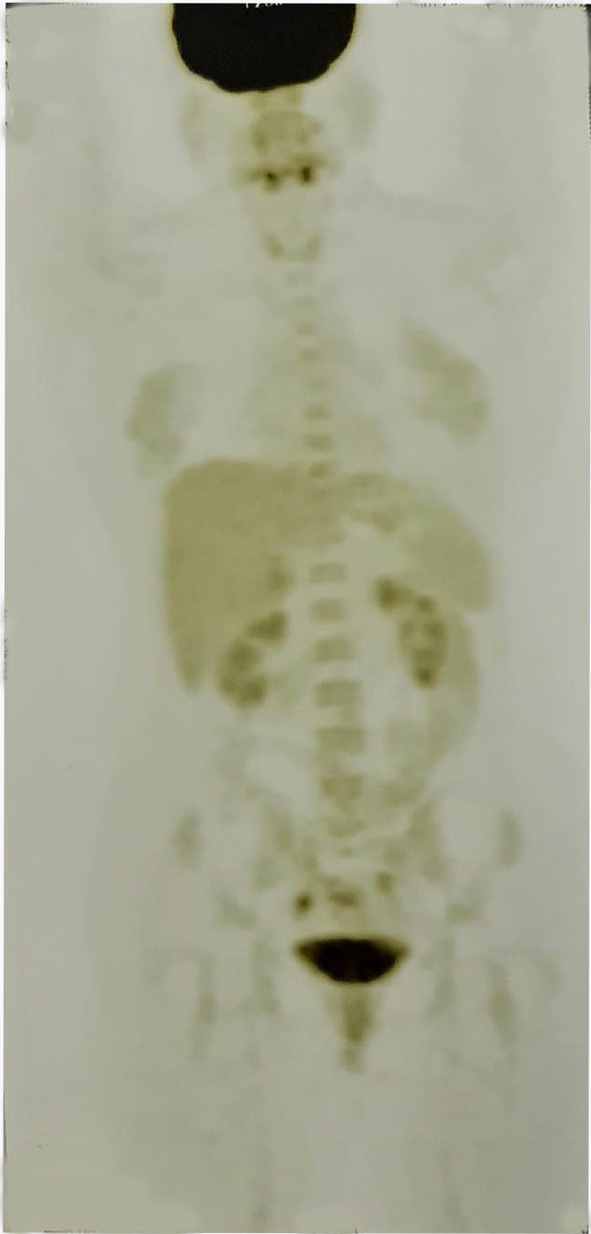
Case no. 1 PET‐scan showing diffuse increased metabolic activity of thyroid gland due to autoimmune thyroiditis.

Initial screening tests, including complete blood count, renal and liver function tests, erythrocyte sedimentation rate, C‐reactive protein, fasting blood sugar, arterial blood gas, urine analysis, and fasting blood sugar, were performed and reported within the normal range (Table 1). Magnetic resonance imaging (MRI) of the brain, thyroid, and abdominopelvic sonography and toxicology tests were also conducted and showed no pathological findings. Cerebrospinal fluid (CSF) analysis was also performed and reported within the normal range. Additionally, the patient's CSF sample was tested for oligoclonal bands, which was negative. However, thyroid peroxidase antibody (TPO‐Ab) levels were significantly elevated, and a positron emission tomography scan (PET‐Scan) revealed diffuse increased metabolic activity in the thyroid gland due to autoimmune thyroiditis (Figure 1).

**TABLE 1:** Initial laboratory data of case no. 1.

Variable	Patient's results	Normal range
White blood cells (*10^3^/μL)	11.2	4–10.5
Hemoglobin (g/dL)	13.4	4.4–5.9
Na (mEq/L)	139	135–150
K (mEq/L)	3.7	3.5–5
Ca (mEq/L)	9.1	8.6–10.3
P (mEq/L)	2.7	2.7–4.7
Mg (mEq/L)	2.5	1.8–2.6
AST (IU/L)	17	<31
ALT (IU/L)	17	<31
AlkP (IU/L)	106	70–380
CRP (mg/L)	7.5	0–10
ESR (mm/H)	19	0–20
VZV‐IgG	<10	<135
VZV‐IgM	0.2	<0.9
Anti‐TPO (IU/mL)	87.7	<5
TSH (μIU/mL)	2.4	0.35–4.94
T3 (ng/mL)	1.3	0.5–1.8
T4 (ng/mL)	5.4	4.8–11.7
HBc Ab	0.16	<1
HBS‐Ag	Non‐reactive	
HCV‐Ab	Non‐reactive	
HIV‐Ag	Non‐reactive	

Based on the patient's medical history, physical examination, and paraclinical findings, AITD associated with focal neurological sign diagnosis was established, and the treatment was initiated. Initially, high‐dose intravenous methylprednisolone (1 g/day) was administered for the first 5 days, followed by 1 g of rituximab on the sixth day. The patient's symptoms significantly improved after starting the methylprednisolone treatment. Additionally, the patient was discharged with oral prednisolone for 30 days, and the patient's condition showed marked progress during follow‐up visits.

### 2.2 Case 2

A 36‐year‐old female patient presented to our clinic with a chief complaint of persistent headache and diplopia. The symptoms had been present for 2 months prior to her initial visit. The patient reported associated symptoms of photophobia, phonophobia, and nausea/vomiting, which prompted her to seek medical attention at another center. The patient reported a sequential progression of visual symptoms, resulting in the development of total ptosis of the right eye, accompanied by diplopia involving both eyes and a blurred visual perception of the left eye. A brain MRI was performed during her first admission, which yielded no pathological findings. However, her initial lumbar puncture revealed an elevation in white blood cell counts (650, lymphocyte:80%, neutrophil:20%) and increased levels of CSF protein (0.1 g/dL). Furthermore, the patient underwent additional evaluations to rule out other differential diagnoses, including Mycobacterium tuberculosis, fungal infections, and malignancies, which yielded no significant findings. A subsequent cerebral angiography did not reveal any significant findings. Following the angiography, the patient developed right‐sided facial palsy and experienced a decrease in hearing in her right ear.

**FIGURE 2:**
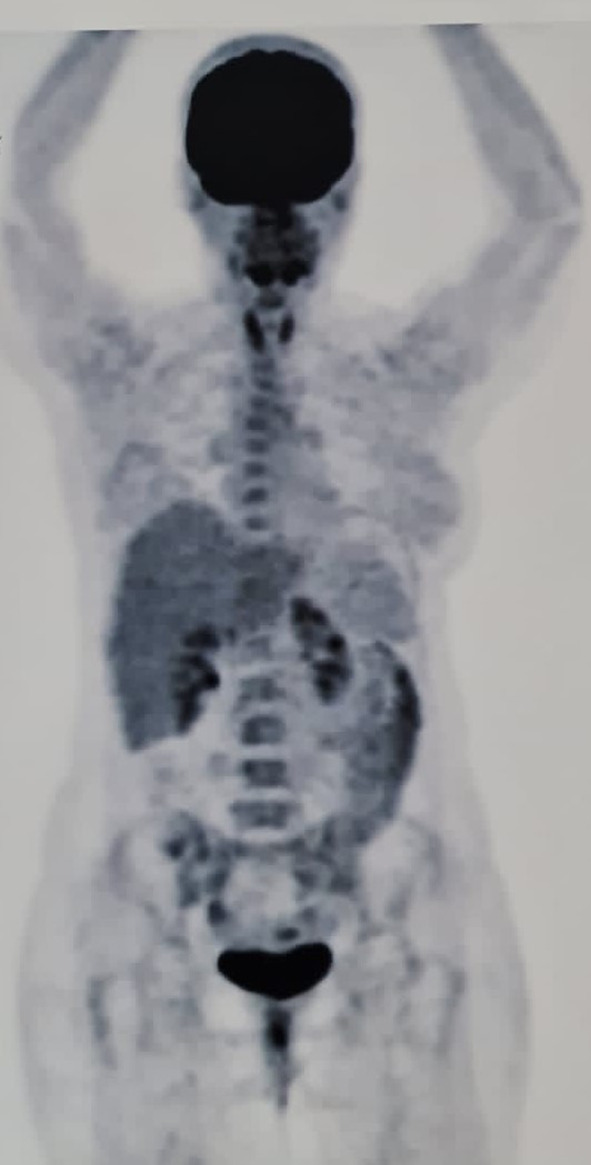
Case no. 2 PET‐scan showing diffuse increased metabolic activity in the thyroid gland without any other significant pathology.

The patient came to our clinic, reporting a progression of the previously mentioned headache, with no improvement in her ophthalmologic issues. Clinical examinations revealed reduced visual acuity in the right eye (finger count at 6 m) and the left eye (reading at 30 cm). The right eye exhibited complete ptosis, with the right pupil non‐reactive to light, while the left eye showed a slow reaction to light. Unidirectional nystagmus was observed in the right eye during right gaze, and the right eye had reduced medial movement. The finger‐to‐nose test for the left eye yielded normal results. Impairment of the XII nerve function was evident, with the patient's tongue deviating to the right side within the mouth and to the left side outside the mouth. Examinations of the V, IX, and X cranial nerves were normal. Furthermore, the tandem gait and deep and superficial sensory examinations were normal. Given the clinical presentation, a new pan‐spinal and brain MRI with gadolinium was requested. The MRI was normal, and the patient's serum IgG4 levels were within the normal range. A PET‐scan was also ordered, demonstrating diffuse increased uptake in the thyroid gland (Figure 2). Laboratory tests of the thyroid gland indicated normal thyroid function (T3, T4, TSH) but positive results for TPO‐Ab (Table 2). Thyroid gland sonography revealed multiple diffused fine hyperechoic nodules. Finally, the patient was initiated on rituximab and low‐dose levothyroxine treatment, which showed a marked improvement in the patient's symptoms.

**TABLE 2:** Case no. 2 thyroid function test results.

Variable	Patient's results	Normal range
TSH (μIU/mL)	2	0.35–4.94
T3 (ng/mL)	1.5	0.5–1.8
T4 (ng/mL)	7.1	4.8–11.7
Anti‐TPO (IU/mL)	727	<34

## 3 DISCUSSION

Autoimmune thyroid disease (AITD) is a widespread autoimmune condition with an estimated incidence and prevalence of between 3% and 5% in people worldwide.^1^ The two primary clinical manifestations of AITD are Hashimoto's thyroiditis (HT) and Graves' disease (GD), with hypothyroidism and thyrotoxicosis serving as the clinical hallmarks of HT and GD, respectively.^4^

AITD is known to have various neurological manifestations, including HE, encephalopathy, coma, depression, psychosis, dementia, cognitive disorders, seizures, sleep apnea, central nervous system (CNS) demyelinating diseases, peripheral neuropathy, ataxia, cerebrovascular accident (CVA)‐like episodes, and headache.^3^, ^5^, ^6^ HE, also known as steroid‐responsive encephalopathy associated with autoimmune thyroiditis (SREAT), is an autoimmune disorder that was first described by Lord Brain et al. in 1966.^7^ HE is characterized by multiple neurological and psychiatric symptoms,^8^ and the diagnosis is based on both clinical and paraclinical findings, such as the presence of aforementioned neurological symptoms, presence of anti‐thyroid antibodies, normal or nonspecific Brain MRI and CSF studies, and subclinical or mild thyroid disease.

In this case report, we describe two cases of young female patients who initially presented with symptoms of focal central neurological disorders associated with AITD, which was not accompanied by HE.

The first patient's clinical presentation of sudden onset left facial plegia initially raised suspicion for a possible CVA. However, further evaluations, including a thorough clinical examination, laboratory tests, and imaging studies, revealed elevated TPO‐Ab, suggesting an underlying AITD. Brain tumors were also excluded from the differential diagnosis list after obtaining an MRI. HE was also ruled out since the patient did not experience neurological symptoms related to HE, including loss of consciousness or mental alterations.

Regarding the second case, the simultaneous onset of persistent headaches and ocular manifestations introduces a myriad of potential differential diagnoses. These encompass cerebral vascular disorders, meningeal irritation associated with infections, space‐occupying lesions, and rheumatological conditions such as sarcoidosis. An elevation in CSF protein levels was noted in our patient. However, this finding may be attributed to blood–brain barrier dysfunction and is expected to resolve completely.^9^ Notably, our patient also exhibited hearing impairment, which has been previously documented in individuals with thyroid disorders, potentially attributable to eustachian tube or middle ear mucosal edema.^10^

As mentioned earlier, AITD has been associated with CNS involvement.^5^, ^6^ The exact relationship between AITD and CNS involvement is not established well. However, some insights can explain the underlying causes of this association. Shared autoantigens may be a potential mechanism underlying the link between AITD and CNS demyelinating diseases. Dysregulation of the immune system may contribute to the development of both conditions and the link between them. AITD and CNS demyelinating diseases have been associated with certain genetic factors, including HLA alleles; therefore, shared genetic factors may contribute to the link between the two conditions.^5^ Additionally, antibodies against CNS tissue and gangliosides were shown to be more common in HT patients than in those with other thyroid conditions, suggesting a possible connection between AITD and CNS involvement.^11^

The treatment for CNS involvement in autoimmune thyroiditis is not well‐defined. However, management of thyroid hormone levels, Immunosuppressive therapy, and symptomatic management can be considered.^5^, ^12^

It is important to note that the diagnosis of AITD‐related functional neurological disorder is one of exclusion, as other causes of CNS involvement, such as viral infections, trauma, and tumors, should be carefully considered and ruled out through appropriate investigations.

In conclusion, AITD‐related focal central neurological disorder is a rare but important manifestation of AITD that can present with different symptoms based on affected nerves. This case report highlights the importance of considering AITD in the differential diagnosis of focal central neurological disorder, particularly in patients with a history of AITD or with suggestive laboratory findings. Prompt diagnosis and appropriate management of AITD‐related functional neurological disorders are essential to prevent long‐term complications. Further research and studies are warranted to better understand the pathophysiology and optimal management of this rare condition.

## AUTHOR CONTRIBUTIONS

**Parisa Sharifi:** Conceptualization; formal analysis; writing – original draft; writing – review and editing. **Sepideh Paybast:** Conceptualization; formal analysis; writing – original draft. **Abdorreza Naser Moghadasi:** Conceptualization; formal analysis; writing – original draft; writing – review and editing.

## FUNDING INFORMATION

The authors received no funding for this study.

## CONFLICT OF INTEREST STATEMENT

The authors declare no conflict of interest regarding this article.

## ETHICS STATEMENT

The current study did not require ethical approval in accordance with local ethical guidelines. The study was conducted in accordance with the Helsinki Declaration, and informed consent was obtained from patients to discuss or publish the details of their disease, their images, and their course of treatment.

## CONSENT

Written informed consent was obtained from patients to publish this report in accordance with the journal's patient consent policy.

## Data Availability

The data used to support the findings of this study are included within the article.
